# Visualization and quantitative analysis of nanoparticles in the respiratory tract by transmission electron microscopy

**DOI:** 10.1186/1743-8977-4-11

**Published:** 2007-11-12

**Authors:** Christian Mühlfeld, Barbara Rothen-Rutishauser, Dimitri Vanhecke, Fabian Blank, Peter Gehr, Matthias Ochs

**Affiliations:** 1 Institute of Anatomy, University of Bern, Baltzerstrasse 2, CH-3000 Bern 9, Switzerland

## Abstract

Nanotechnology in its widest sense seeks to exploit the special biophysical and chemical properties of materials at the nanoscale. While the potential technological, diagnostic or therapeutic applications are promising there is a growing body of evidence that the special technological features of nanoparticulate material are associated with biological effects formerly not attributed to the same materials at a larger particle scale. Therefore, studies that address the potential hazards of nanoparticles on biological systems including human health are required. Due to its large surface area the lung is one of the major sites of interaction with inhaled nanoparticles. One of the great challenges of studying particle-lung interactions is the microscopic visualization of nanoparticles within tissues or single cells both *in vivo *and *in vitro*. Once a certain type of nanoparticle can be identified unambiguously using microscopic methods it is desirable to quantify the particle distribution within a cell, an organ or the whole organism. Transmission electron microscopy provides an ideal tool to perform qualitative and quantitative analyses of particle-related structural changes of the respiratory tract, to reveal the localization of nanoparticles within tissues and cells and to investigate the 3D nature of nanoparticle-lung interactions.

This article provides information on the applicability, advantages and disadvantages of electron microscopic preparation techniques and several advanced transmission electron microscopic methods including conventional, immuno and energy-filtered electron microscopy as well as electron tomography for the visualization of both model nanoparticles (e.g. polystyrene) and technologically relevant nanoparticles (e.g. titanium dioxide). Furthermore, we highlight possibilities to combine light and electron microscopic techniques in a correlative approach. Finally, we demonstrate a formal quantitative, i.e. stereological approach to analyze the distributions of nanoparticles in tissues and cells.

This comprehensive article aims to provide a basis for scientists in nanoparticle research to integrate electron microscopic analyses into their study design and to select the appropriate microscopic strategy.

## 1. Introduction

Each day a human inhales and exhales more than 10,000 litres of air. With an epithelial surface area of approximately 140 m^2 ^[1], the internal surface of the human lungs is destined to interact with an enormous number of airborne particles with each breath. After inhalation particles encounter several protective structural and functional barriers of the respiratory tract which include the surfactant film [2-4], the aqueous lining layer with the mucociliary escalator [5], airway and alveolar macrophages [6-8], the epithelium with the underlying basement membrane [9,10], and dendritic cells residing in or underneath the epithelial layer [11].

Particles can be classified according to their size which predominantly defines to which compartments of the lungs they gain access [12]. In recent years, particulate matter at least in one dimension smaller than 100 nm has become a focus of pulmonary particle research [13-19]. For the purpose of this review, particles < 100 nm at least in one dimension will be referred to as nanoparticles (NP) although the authors are aware that several practical subclassifications of particles exist [20], e.g. with respect to their shape (tubes, rods etc.) or their origin (combustion-derived nano-sized particles are usually referred to as ultrafine particles to distinguish them from synthetic NP). The growing interest in NP has several obvious reasons. First of all, there is epidemiological evidence that the nano-sized fraction of particles associated with air pollution is a major contributor to adverse health effects attributed to air pollution [21,22]. Additionally, a growing number of experimental studies have focussed on the enhanced toxicological potential of synthetic NP in contrast to larger sized particles of the same material [23,24]. While the progress of nanotechnology basically relies on the fact that NP may have different physicochemical properties than larger sized particles of the same material, it has been recognized that these different features may also be accompanied by a different biological reaction of the cells of the respiratory tract upon exposure [25,26]. Although this fact clearly poses a challenge to researchers involved in pulmonary toxicology, it is also of particular interest for respiratory medicine since inhaled NP may offer an innovative approach for an improved medical treatment [27].

Where then is the justification to employ different transmission electron microscopic (TEM) tools for the analysis of the interaction of NP with cells of the respiratory system? First of all, it is of principal interest to investigate whether the morphology of the tissues and cells of interest changes following NP exposure. Usually, conventional light and electron microscopic methods will suffice to address these questions. Second, however, the localization and distribution of particles within tissues and cells needs to be studied to understand how and why NP cause cellular responses and whether a targeted particle has reached its target cell compartment [28,29]. Unfortunately, NP may not always be distinguishable from cellular organelles by conventional TEM which evokes the requirement of analytical microscopic methods such as energy filtered TEM (EFTEM) [16,30]. On the other hand, when the entering mechanisms of particles into cells are under investigation it may be necessary to distinguish between cellular compartments that cannot be identified by their morphological appearance alone (e.g. early and late endosomes). Immunogold labeling of the compartments may help to overcome these limitations. Since all structures including NP present as a two-dimensional profile on a tissue section, a high resolution 3D microscopic approach by electron tomography is desirable to study the interaction of NP with cellular organelles as well as their 3D shape and size after contact with the cells [31]. Importantly, it will be necessary to analyze the morphological alterations of the lung (e.g. investigate potential emphysema development upon long-term NP exposure) and the distribution characteristics of NP in pulmonary tissues and cells in appropriate quantitative terms for which stereology offers a great number of unbiased and efficient methods [32-34].

Though the importance of TEM analyses in NP research is unquestionable the use of advanced imaging techniques and appropriate quantification is still rare. We are convinced that this may partly result from the unawareness of the pitfalls when applying conventional TEM to NP research and the possibilities to overcome these problems. Despite the great efforts to standardize and optimize the generation and exposure systems of NP [35] the appropriate application of microscopic techniques requires optimization. Therefore, this review aims to provide a comprehensive overview of advanced TEM techniques and a critical appraisal of their potentials and limitations in order to stimulate the implementation of more advanced and quantitative TEM methods for NP-related research in the respiratory tract.

## 2. Focus A: Transmission electron microscopic methods

### 2.1. Fixation and embedding

**Figure 1:**
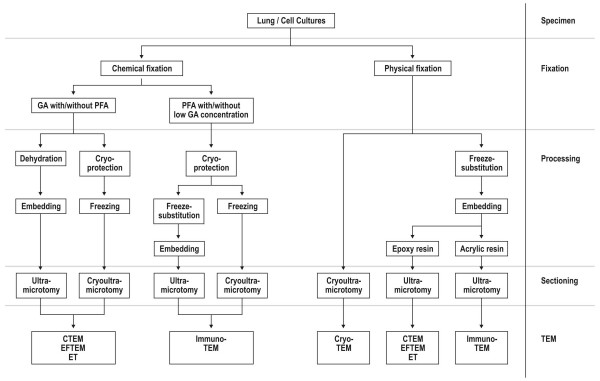
**Survey on several transmission electron microscopy strategies**. The figure provides help to chose a specific strategy for TEM preparation based on the scientific purpose of the study. It shows the crucial decisions in specimen preparation from the fixation level to the investigation at the TEM. Abbreviations: GA = glutaraldehyde; PFA = paraformaldehyde; CTEM = conventional TEM; EFTEM = energy-filtered TEM; ET = electron tomography.

**Figure 2:**
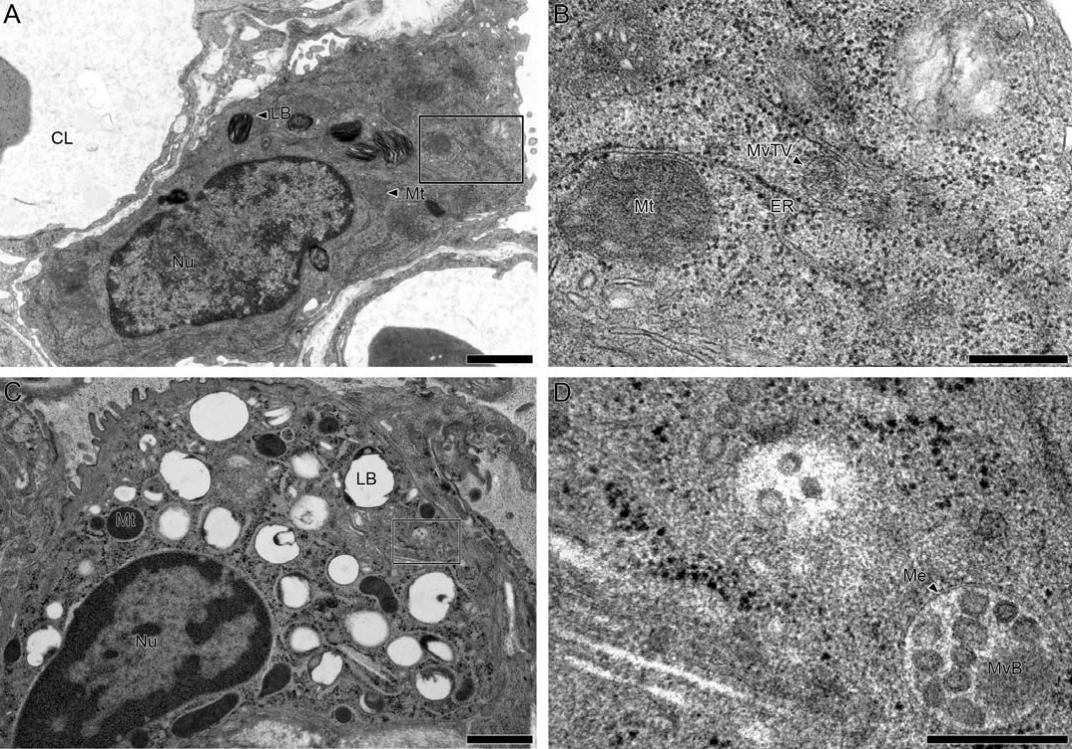
**Chemical and physical fixation of the lung**. Alveolar epithelial type II cells were studied by cTEM either after chemical (A and B) or after physical (C and D) fixation. The overview in A shows a well-preserved type II cell from a newborn rat lung fixed by instillation of 1.5% GA, 1.5% PFA in Hepes buffer and processed according to Table 1. Lamellar bodies (LB), nucleus (Nu) and mitochondria (Mt) are well preserved. At a higher magnification, details of the endoplasmic reticulum (ER) as well as an ER related multivesicular transport vesicle (MvTV) can be visualized. The overview in C shows a well-preserved type II cell from an adult rat lung. A small piece of tissue was cut from the whole lung, put in a syringe with 1-hexadecene and air was extracted from the tissue block by negative pressure. Afterwards, the specimen was high-pressure frozen (Leica EMPact 2.0, Leica, Vienna, Austria), freeze-substituted with acetone containing 1% osmium tetroxide (AFS 2.0, Leica, Vienna, Austria) and embedded in epoxy resin. Most likely due to the lack of uranyl acetate during freeze-substitution the lamellar bodies are not well preserved, with almost complete loss of the surfactant material, only the limiting membrane can be seen. However, the ultrastructure of other organelles like multivesicular bodies (MvB) is highly increased (D) due to the excellent preservation of the membrane structures (Me). Since this is the first description of high-pressure frozen lung tissue, systematic studies are needed to determine the ideal processing both for conventional and immuno TEM. Bars = 1 μm (A, C), 250 nm (B, D).

A prerequisite for transmission electron microscopy is that all material entering the microscope has to be fixed in one way or another. Fixation of cells and tissues aims to preserve the specimens as close to the living state as possible. As further outlined, different electron microscopic techniques as well as specific questions of a particular study significantly influence the choice of the fixation and embedding method. Currently, there are two major approaches to fix biological samples, viz. chemical or physical fixation. For the lung as an entire organ, there is no routine alternative approach to chemical fixation to date but for restricted tissue samples, such as larger airways or cell cultures, physical fixation offers an excellent alternative. The following paragraphs as well as Figure 1 provide an overview on different possible methods, their impact for the different TEM techniques and relevant references. Nevertheless, one will have to evaluate the usefulness of a specific protocol for each particular study. For this reason, Weibel et al. [36] have introduced a number of very instructive external and internal standards. In Figure 2, we provide a chemically and a physically fixed specimen for comparison between both methods.

#### 2.1.1. Chemical fixation

Two basic routes exist for the delivery of chemical fixative to the lung: instillation via the airways [1,37] and perfusion through the vasculature [38,39]. Instillation fixation via a cannula introduced into the trachea is a straightforward method to fix the lungs, however, it disturbs the surface lining ultrastructure of airway or alveoli including mucus, surfactant, macrophages and foreign material as NP. The lungs need to be collapsed prior to instillation of the fixative at a pressure of approximately 20–25 cm of water. Afterwards the trachea is clamped to prevent fluid outflow, the lung is removed from the chest cavity and stored in cold fixative, e.g. for at least 24 hours [40]. These procedures guarantee that the lungs are fixed at around two thirds to three fourths of total lung capacity [41].

Vascular perfusion fixation via a cannula/catheter introduced into the pulmonary trunk fixes the lungs very quickly and thoroughly [42], but its performance is far more complex and demanding than instillation [43]. Air and perfusate inflow pressures need to be in a correct relation to each other, controlled and possibly adjusted throughout the fixation procedure in order to obtain the tissue of the whole lung properly fixed. This is in itself technically demanding. Particular attention must be paid to the question of which effective perfusion pressure should be used. Since the intrapulmonary pressure can be adjusted the lung can be fixed at a nearly arbitrary fraction of total lung capacity with excellent results at approximately 60% of total lung capacity. A detailed instruction and discussion of both methods is provided by Gil [39] and Gehr et al. [41].

First, the preferred method of fixation for NP research depends on the actual scientific question. If the uptake of particles by alveolar macrophages or their interaction with surfactant is the subject of interest one will have to use vascular perfusion fixation. However, if the translocation of NP to the blood circulation is investigated it will be desirable not to wash out all the blood from the pulmonary circulation and one would therefore prefer to use instillation fixation. Second, however, the choice of the fixation method is also influenced by the experience and skills of a laboratory. If one is not familiar with the different aspects of vascular perfusion fixation and their interpretation this technique may lead to false conclusions because the relationship between the airway and vascular pressures determines the structural appearance of the parenchyma [43].

Fixation of lung samples by immersion in the fixative is by far the least preferable technique because the lung tissue is collapsed and artefactual changes occur, which can not be controlled for [44]. It should therefore only be applied when neither perfusion nor instillation fixation can be used (human biopsy material or specimens sampled from one individual over a sequence of time). In cell cultures, immersion fixation is the method of choice.

The fixative consists of a fixing agent in a suitable vehicle [45]. The most widely used fixatives contain paraformaldehyde (PFA) and glutaraldehyde (GA) often combined with a post-fixative, mostly molecules including heavy atoms such as uranium or osmium [44,46]. PFA is a higher polymer of formaldehyde and is sold as a white powder. Formaldehyde solutions made of PFA do not contain methanol in contrast to the 10% formalin solutions frequently used in light microscopy [47]. There is a large number of protocols using either PFA or GA individually or a combination of PFA and GA, in varying concentrations and with different buffers. Often, the choice of the fixation solution is not based on scientific reasons but on lab traditions. Detailed reviews of the literature on this topic are available [48-50]. The choice of the fixative as well as the further processing of the samples will depend on the actual purpose of the study because chemical fixation always means a compromise between preservation of cellular ultrastructure and maintenance of antigenicity. High concentrations of GA as well as osmium containing post-fixatives often delete the antigenicity and make samples unfeasible for immuno TEM studies [48] whereas very low GA concentrations as well as the lack of exposure to uranyl salts fail to sufficiently preserve pulmonary ultrastructure [51]. In Table 1, we recommend two protocols for chemical fixation and embedding for conventional and immuno TEM of the lung routinely used in our lab though we are aware that many other suitable protocols may exist.

**Table 1:** Two protocols for conventional and immuno TEM preparations routinely used in our lab.

	**cTEM**	**Immuno TEM**
**Fixative**	1.5% GA, 1.5% PFA in 0.15 M Hepes buffer	0.1% GA, 4% PFA in 0.2 M Hepes buffer
**Postfixative**	1% osmium tetroxide in sodium cacodylate buffer	---
**En-bloc staining**	Half-saturated aqueous uranyl acetate	0.5% uranyl acetate in methanol (at -90°C)
**Dehydration**	Ascending acetone series	Methanol (at -90°C)
**Embedding medium**	Araldite (at 60°C)	Lowicryl HM 20 (at -45°C under UV light)

Note. In this table two different protocols of specimen preparation for cTEM and immuno TEM, respectively, are given. Detailed descriptions of the protocols are given in Fehrenbach and Ochs [51]. Abbreviations: GA = glutaraldehyde, PFA = paraformaldehyde.

#### 2.1.2. Physical fixation

Physical fixation is often referred to as physical immobilization, cryo-immobilization or cryofixation. The purpose is to solidify the water in the sample, thereby arresting the biological machinery. However, the formation of ice crystals induces severe damage to the cell [52]. Cryo-immobilization has a long-standing history in light microscopy since segregation artefacts, induced by ice crystals reside in a size range of about 100 nm, well below the resolution limit of light microscopic techniques.

In order to be useful for TEM studies, these segregation artefacts need to be prevented, or reduced below the resolution limit of the TEM. This can be achieved by vitrification: a phase transition of liquid water to the vitrified state, which is an amorphous (i.e. lacking crystalline structures), solid state of water [53-57]. Such a state of water can only be achieved by extremely fast extraction of heat from the sample surface [58]. As water is a poor thermal conductor and the most abundant molecule in most biological samples, sufficient heat can be eliminated only from small samples [59]. In fact, after plunging the average biological sample in a cryogen (e.g. liquid nitrogen), a superficial layer of about 10–20 μm is vitrified but the ultrastructure of deeper structures will be damaged by ice crystal growth [60]. Therefore, applications involving small particles (e.g. viruses or protein complexes) or single cells were particularly attracted by this technique [61-63].

It has been recognized that high pressure (roughly 200 MPa) influences the physics of water in a way cryoprotectants do [64] which led to the development of high pressure freezing (HPF) procedures [58,65,66]. Application of 200 MPa pressure during the cooling process increases the depth of vitrification ten-fold [50,67,68], thereby making physical immobilization available up to 200 μm and suitable for larger biological objects, such as tissues [69-72] and even entire multicellular organisms [73]. The impact of 200 MPa on the survival rate of cellular organisms was shown to be minimal [65,74] but not absent [75,76].

The vitrification of the water in the sample results in a highly increased preservation of the fine structure and an improved retention of the structural components compared to chemical fixation [61,66,77-79] and is therefore often superior for antigenicity studies at the TEM level [80-82].

After successful vitrification, a variety of follow-up procedures are available (Figure 1). The fully hydrated specimen can be cryosectioned and investigated under a cryoTEM [56,69,83]. As no dehydration step takes place, this approach will give the closest impression of the native biology of the sample. The topology of the sample can be studied by freeze fracturing methods [84,85].

Alternatively, the water can be removed from the sample either by freeze drying [86,87] or by the much more widely used technique of freeze substitution [88]. Dehydration of the sample implies that the threat of ice crystal damage does not longer exist. Low temperature embedding became available after the development of resins with a low enough viscosity to allow infiltration and polymerization at temperatures below 0°C [82,89,90]. However, since the sample can be returned to room temperature conditions, the techniques established for chemically fixed samples apply as well.

We here for the first time present evidence that lung tissue can be fixed by HPF (Figure 2). However, as air-filled spaces in the lung cannot withstand the pressure induced (about 200 MPa) an approach is required in which the airways must be filled with an inert, uncompressible fluid, e.g. perfluocarbon, prior to freezing.

### 2.2. Conventional TEM

**Figure 3:**
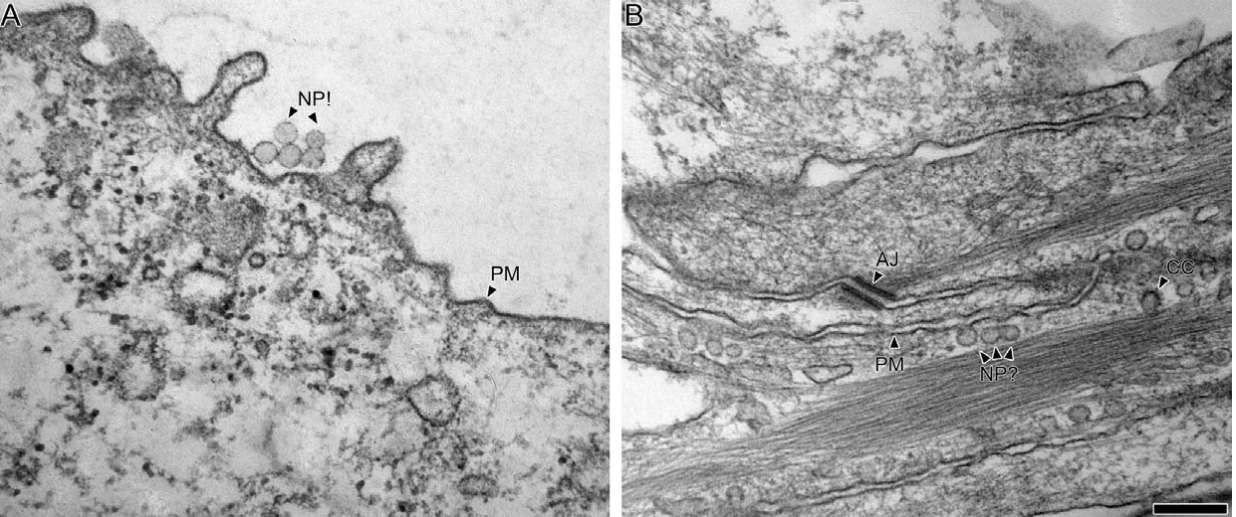
**Conventional TEM of polystyrene nanoparticles**. This figure demonstrates the impossibility to distinguish between NP and cellular structures by conventional TEM unambiguously. In A, five polystyrene NP (NP!) with a mean diameter of 78 nm are observed next to an A549 cell. Once taken up by the cells, they may have an appearance as shown in B. It is very likely that the spherical structures in B (NP?) are not NP but vesicular structures like caveolae. CC = Clathrin coated vesicle; PM = Plasma membrane; AJ = Adherens junction. Chemical fixation, Epon embedding, 40–70 nm sections. Bar = 1 μm (A and B are at identical magnification).

Conventional TEM in NP research is frequently used to characterize particle structure [18,91-93], to demonstrate the intracellular localization of NP [28,94-96] and less frequently to assess the morphology of tissue or cell samples [28,96]. Its popularity is partly explained by the high resolution and because it is established in many laboratories. However, particles that are shown in TEM figures are often agglomerated structures with diameters of far more than 100 nm. For this reason, too low magnifications are used, which do not allow the identification of particles in the range of 10–20 nm or even less. Furthermore, it is often ignored that NP can sometimes be indistinguishable from cellular structures in the same size range. For example, electron dense particles (e.g. titanium dioxide) may have similarity with glycogen granules, mitochondrial matrix granules or ribosomes whereas electron lucent particles (e.g. polystyrene) may be confused with spherical vesicular structures [97]. Therefore, there is a potential risk that a technical bias is present either due to the fact that cellular structures are mistaken for NP or vice versa. Using only conventional TEM this bias cannot be overcome (Figure 3).

Despite its wide use in NP research, conventional TEM as a sole tool for the qualitative or quantitative evaluation of the cellular localization of NP needs to be carefully evaluated from case to case. With few exceptions (e.g. colloidal gold particles), it needs to be accompanied by methods that increase the identification of every NP, independent of its size, shape or electron transmissibility. Nevertheless, the application of conventional TEM is justified by its suitability to detect potential effects of NP on cellular ultrastructure and to characterize NP structure.

### 2.3. Immuno TEM

Once a specimen has undergone all fixation and embedding procedures, it has maintained its antigenicity, and preserved its ultrastructure, immuno TEM offers some useful applications for NP research: visualization, co-localization and quantification of NP and antigens [98-101]. However, the authors are not aware of any study related to NP that used immunocytochemistry and TEM. The purpose of immunogold labeling is to localize pools of proteins and lipids or to identify structures that cannot be clearly identified or cannot be seen at all at high resolution. For example, a primary antibody binding to the antigen of interest is visualized by a secondary antibody carrying a colloidal gold particle of defined size. This gold particle is visible under the TEM due to its electron density. In contrast to light microscopy, this method allows to determine the localization of antigens in association with subcellular structures at the nano-meter range.

If NP could be made visual by immunogold labeling their identification would be straightforward and unambiguous. The binding specificity could then be cross-checked by the co-localization of the particle (which cannot be performed with immunogold labeling of proteins). Once particles are undoubtedly detectable by TEM, it is possible to determine their exact localization within cellular organelles and to quantitatively assess their tissue or cellular distribution in an unbiased way (see below, stereology). This approach requires that the particles have a surface coating which can be recognized by a specific antibody. As the surface coating is likely to influence the biological effects of the NP, this approach for NP detection might not be advantageous in toxicological studies. However, particles targeted to specific tissues, cells or organelles are designed with a specific coating that guides them their way. In these cases, immunogold labeling for detection may be feasible and very useful.

**Figure 4:**
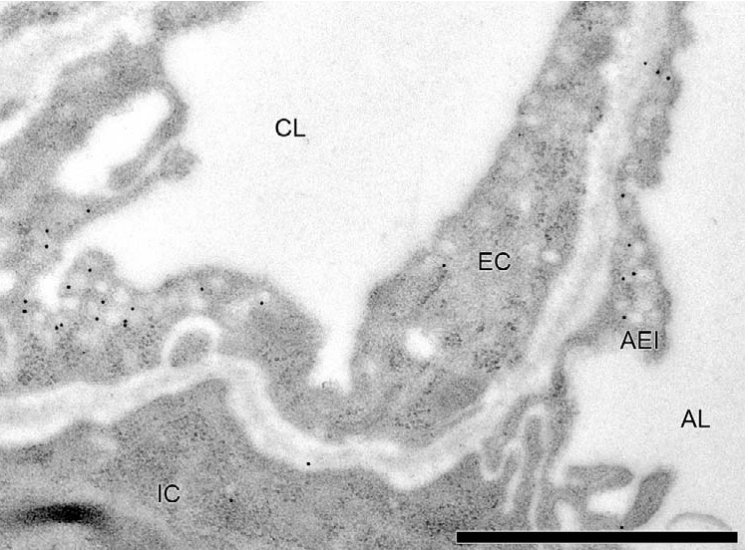
**Immuno TEM of rat lung labeled for caveolin-1**. Caveolae are cholesterol-rich regions of the plasma membrane involved in endocytosis. One of the constituting proteins of caveolae is caveolin-1 which was labeled here using newborn rat lung tissue fixed by instillation of 4% PFA, 0.1% GA in 0.2 M Hepes buffer. After freeze-substitution and embedding in acrylic resin (Table 1), ultrathin sections (40–70 nm) were cut and mounted on formvar-coated Ni mesh grids. Immunogold labeling was performed according to standard protocols [99]. The primary antibody was a rabbit anti-caveolin-1 antibody (BD Biosciences, Pharmingen, Germany) diluted 1:50. The secondary antibody was a goat-anti-rabbit antibody coupled to 10 nm gold particles (British Biocell, Cardiff, United Kingdom). A strong signal is found for caveolae in capillary endothelium and alveolar epithelium. Unspecific background labeling was weak (note the gold particle in the interstitium) but not completely absent. Immunogold labeling requires good knowledge about the biology of the target antigen and the specificity of the antibody. Before going to the TEM level, one is well advised to perform pilot light microscopic experiments. CL = Capillary lumen; EC = Endothelial cell; IC = Interstitial cell; AEI = Alveolar epithelial type I cell; AL = Alveolar lumen. Bar = 1 μm.

If coated NP are labeled by immunogold particles it may be interesting to determine their exact localization. For example, early and late endosomes as well as lysosomes cannot be distinguished from each other by their morphology alone. The double-labeling technique (e.g. using 5 nm golds for the NP and 15 nm golds for the organelle) may represent an interesting approach to perform co-localization studies. In those cases where NP can be identified by EFTEM (see below) immunogold labeling of organelles or cytoskeletal proteins offers a detailed analysis of how NP are associated with cellular proteins. Finally, exposure of cells to NP may result in alterations of the cellular distribution of a particular antigen [102] which can be studied by quantitative immuno TEM [103]. An example of sufficient immunogold labeling for an organelle suspicious of being involved in active cellular NP uptake is shown in Figure 4.

### 2.4. Energy filtered TEM

As shown above, the major problem in the investigation of interactions between NP and the lung is the fact that it is often not possible to distinguish unambiguously between cellular structures and NP, a fact that is often neglected or even unrecognized. Energy filtered TEM combines the high resolution of the TEM with the analytical capabilities of electron energy loss spectroscopy and imaging [104]. This allows the analysis and visualization of the spatial distribution of the elemental composition of a particular structure. During transmission of the beam, inelastic scattering events between beam electrons and the specimen occur, resulting in a specific loss of energy of the electrons in the beam. In such a way, the atoms in the sample leave their fingerprint in the transmitted electron beam. By analysing the energy spectra of the transmitted electrons, the elemental composition of the atoms in the specimen can be retrieved. However, EFTEM requires the use of sections with a thickness of about 30 nm in order to avoid multiple scattering [105].

NP often contain elements such as titanium, zinc or cadmium with no or low abundance in biological systems. This differential atomic composition allows for the distinguishing potential of elemental mapping, and therefore of the NP. If lungs or cell cultures are exposed to an aerosol containing NP of a known composition, it is possible to test whether and which cells or organelles contain the elements the NP are composed of.

EFTEM is an advanced imaging technique that requires substantial technical know-how and a suitable microscope, which explains why it is not widely established in life sciences. If the elemental composition of the particles is not known beforehand, the analysis by electron energy loss spectroscopy may turn out cumbersome and frustrating. Furthermore, analysing NP within biological preparations using EFTEM may also be frustrating if the NP consists of elements that occur in large amounts in biological systems as well although recent progress in the EFTEM technique might improve this situation [106]. Electron spectroscopy images should always be accompanied by electron energy loss spectra of the region of interest and a negative control region (e.g. pure embedding media).

So far, the use of EFTEM in pulmonary research is limited to a few studies, e.g. analysing plasma membrane integrity by lanthanide tracers [107] or phosphorous spreading in alveolar type II cells during ischemia [108,109]. Research involving the intracellular localization of NP at high resolution should adopt more frequently the use of this methodology. Although the authors are aware that EFTEM requires a great deal of resources (highly trained personnel and infrastructure) the effort seems justified by the fact that there is no real alternative, especially if particles are subject to quantification of their distribution.

**Figure 5:**
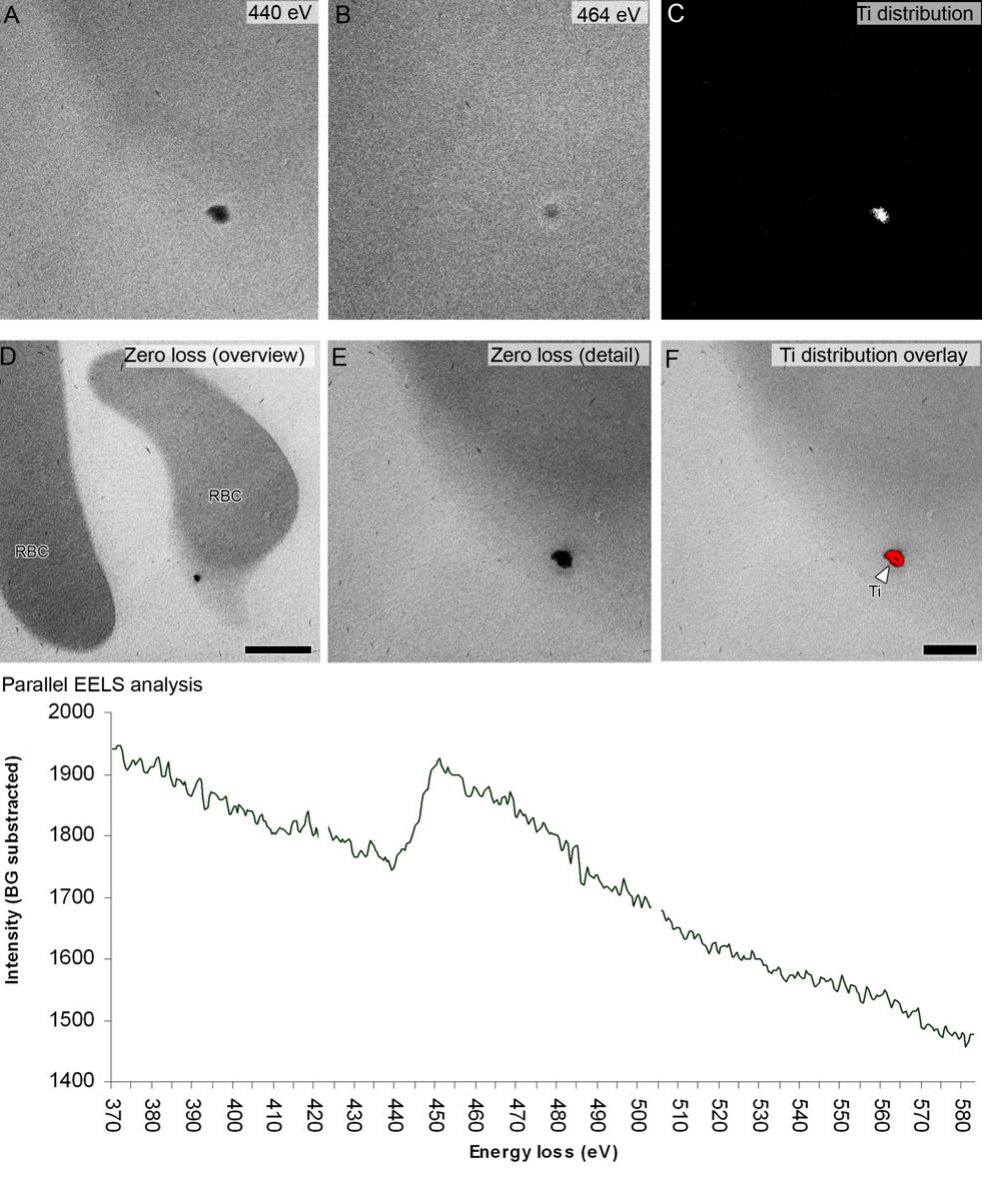
**Titanium detection by ESI and pEELS**. A and B show an energy loss imaging (ESI) series of the L3 orbital of Ti at 440 eV (background) and 464 eV (signal) in an erythrocyte culture. In C, the resulting difference is calculated, revealing the distribution of Ti in the sample. Not shown, but used in the three-window calculation of the Ti distribution is a second background window recorded at 390 eV. The occurrence of Ti is confirmed by parallel electron energy loss spectroscopy (pEELS), shown in the graph. The energy a beam electron looses when interacting with the sample is representative for the atom and orbital it is interacting with. The graph shows a peak intensity around 460 eV, indicative for the interaction with the L3 orbital of Ti (the background follows a negative exponential progression). The zeroloss overview and detail images show a dark particle near a red blood cell. With pEELS confirming the occurrence and ESI revealing the distribution, the particle can be appointed as containing Ti. Bars = 1 μm (D), 250 nm (A to F except D). Titanium dioxide particles were incubated with erythrocytes and fixed and embedded conventionally. The section was placed on a Ni-grid. No supportive film and no staining were used. Zeroloss measuring of the relative thickness of the section at 10 different positions revealed a t/λ of 0.38 (+/- 0.04).

Fortunately, EFTEM has witnessed a more frequent use in recent years for the study of NP localization in vitro [16,95], in vivo [14,30,110,111] and in sputum macrophages [112]. Yet, the majority of the ultrastructural studies still relies on only conventional TEM images without additional proof of the particle identity. In Figure 5, we provide an example of titanium dioxide NP visualized by EFTEM.

### 2.5. Electron tomography (ET)

Understanding the interactions of NP with cellular structures, especially with complex membrane-bound organelles requires information about the three-dimensional (3D) arrangement of the morphological components. Before the development of ET, high resolution 3D reconstruction was based on a sequence of serial sections [113-115], which was hampered by the fact that delicate membranous structures would require the production of sections as thin as 5–10 nm, which is not feasible due to limitations in ultramicrotomy. Since in TEM the entire thickness of the section is constantly in focus, the z-resolution of 3D reconstructions from serial sections is limited by the section thickness. In NP research, the use of serial sections is even less sufficient because the diameter of the particles under investigation is often smaller than 50–70 nm, the usual thickness of an ultrathin section.

The development of ET has made it possible to overcome these problems: A rotational relationship between detector (camera) and object is created by tilting the section inside the electron microscope at user defined increment intervals, usually 1° or 2°. This gives rise to a tilt series: a sequence of projections of the object, taken from different angles [116]. Subsequently, the tilt series undergoes several automated digital processing steps including alignment and backprojection [117] by which the object of interest is reconstructed in its three dimensions. The result is a stack of parallel slices, with an axial resolution (distance between two subsequent slices) in the range of 2 to 10 nm, i.e. several times thinner than the thinnest section one could possibly generate physically. Additionally, volume and surface rendering software may be applied to obtain an enhanced 3D impression [118]. In a process known as segmentation [119], particular structures of interest can be extracted, either automated or manually, for further analysis and visualization. Importantly, the segmentation process is influenced by the observer interpretation of the ET slices and therefore the result of a selective cognitive process.

Due to the nature of the electron beam and the increasing relative thickness of the section at increased tilting angles, the section can only be inclined between about ±70°. The remainder, between ±70° and ±90° (the so-called missing wedge) cannot be recovered from the sample and will result in artefact formation during digital reconstruction [120].

For the moment, ET is not widely established for several reasons. First, the electron microscope must be equipped with a motorized goniometer and specialized software, and such instrumentation is resourceful, which, in turn currently limits their purchase to a number of central TEM labs worldwide. Second, ET requires advanced experience and know-how in electron microscopy, specimen preparation and interpretation of the TEM images. The principles and problems of ET are discussed in a number of excellent reviews [121-124].

ET is only emerging to be part of the methodological repertoire in NP research and has already been applied on nanomaterials [125] as well as initial studies on the interactions of NP with biological structures [31,126].

**Figure 6:**
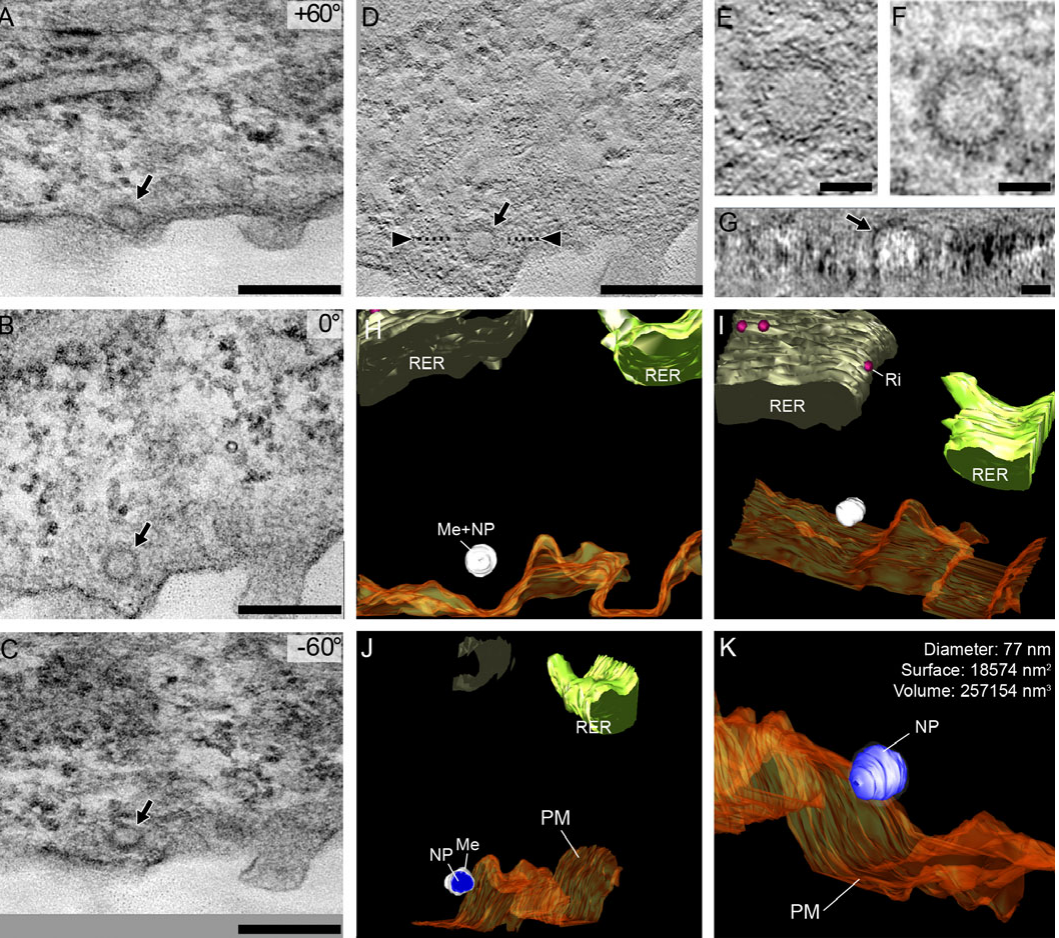
**Electron tomography of a 250 nm thick section**. A tilt series (three stills are shown in A, B and C) between +/- 60° with an increment of 1° provides the information for a volume reconstruction by weighted backprojection (D) of an in vitro grown alveolar epithelial cell (cell line A549), exposed to polystyrene NP prior to chemical fixation. The 2 nm thin slice (E, at a depth of 58 nm in the section) reveals a crisper and clearer depiction of the polystyrene NP than the 250 nm thick Epon section (F). Arbitrary digital slices can be made (G, position shown by the two arrowheads in D) in order to provide unquestionable recognition of the NP. Bars = 250 nm in A-D, 50 nm in E-G. Software based 3D rendering offers a way for segmentation according to the interpretation of the user and allows full perspective freedom (H and I). Moreover, clipping planes can partially dissect the scene (J), segmented objects can be omitted (the membrane surrounding the NP in K) and specific quantitative information on rendered objects obtained. Blue: nanoparticle, shades of green: rough endoplasmic reticulum (RER), orange: plasma membrane, transparent white: membrane surrounding the NP, red: ribosomes (Ri) on the RER. Me = membrane, NP = nanoparticle, PM = plasma membrane.

Visualization by ET allows the analysis of NP shape, volume and surface in 3D. This greatly helps with the characterization of NP and the discrimination between genuine particles and agglomerated NP. Furthermore, ET is projected to become a useful tool to study contact sites between NP and macromolecules in detail. Such insights will potentially improve our understanding of NP entry mechanisms into cells, processing by the cellular machinery and where NP related toxicity originates. One exciting topic also relates to the fact that ET extends the application of a number of stereological tools (e.g. the optical disector, see below) to the electron microscopic level [127]. Particularly, for stereological purposes using ET no segmentation process is needed making the quantification independent of the observer preference. Finally, it needs to be emphasized that ET is independent of sample processing: it can be carried out on materials embedded in epoxy or methacrylate resins as well as cryosections and fully hydrated cryosections and allows to be combined with immuno TEM. Figure 6 provides an example of a 3D reconstruction of a NP in its cellular environment.

## 3. Focus B: Correlative light and electron microscopy

In an ideal world, it would be possible to investigate the interactions of cells with NP combining the benefits of light microscopy, especially live cell microscopy, with the high resolution of transmission electron microscopy. However, in laser scanning microscopy the resolution is limited to 200 nm and 500–900 nm in the lateral and axial directions, respectively, although deconvolution algorithms may increase the resolution 2–3 fold [16,128,129]. On the other hand, with TEM only fixed specimens can be analyzed. A combination of light and electron microscopy gives the most complete and accurate picture. Correlative light and electron microscopy is particularly useful for the interpretation of light microscopic data because the low resolution often limits the conclusions that can be drawn from light microscopic investigations alone [130]. To overcome the gap between light and electron microscopy several approaches have been developed which might prove to be very helpful to correlate light and electron microscopic observations in NP research.

Two highly promising ways for correlative light and electron microscopy have been described in detail recently. Using a linker molecule, the fluorescent signal recorded by the optical system of the light microscope is converted into a signal that can be picked up by the optical system of the TEM. In a first approach, a biotinylated antibody specifically directed against a particular antigen is applied and visualized with a streptavidin labeled quantum dot that contains fluorescent molecules at the surface and an electron dense core (CdSe) which can unambiguously be detected by EFTEM [131,132]. Nisman et al. [132] demonstrated this method by localization studies of the nuclear promyelocytic leukaemia protein in the nucleus of cultured cells. Quantum dots can be quantified similarly to immunogold particles but they are less distinct than colloidal gold particles. A second approach has been described by Griffin et al. [133] and makes use of a short tetracysteine hairpin motif that can be integrated into a large number of proteins. The cells can then be exposed to a biarsenical fluorescent dye which is able to permeate through cell membranes and spread a strong fluorescence signal upon contact with the tetracysteine motif. If diaminobenzidine is added and strong illumination is applied, the biarsenical group reacts with the diaminobenzidine, a process known as photo-oxidation. The diaminobenzidine reaction product is strongly osmiophilic and can easily be visualized by TEM. Gaietta et al. [134] have used this method to visualize the gap junction protein connexin 43 in HeLa cells at light and electron microscopic level.

Direct ways denounce the use of linker molecules between the two microscopic modes. The idea rests on the comparison of images obtained by light and and electron microscopy of the same region of interest. In the case of labeling of specific probes, the signal is obtained by light microscopy (either a precipitation or a fluorescent signal) and superimposed on the high resolution micrographs generated by TEM. One simple way is to use a low-temperature embedded or frozen specimen and cut consecutive ultrathin sections, which can be used for immunofluorescence imaging and immunogold labeling, respectively [135,136]. This approach allows to visualize "almost the same" region within a specimen, which, however, is not sufficient for studying the "spot-like" interactions of NP with cellular structures. It is therefore desirable to view exactly the same structure of interest at light and electron microscopic level. An interesting approach has been described by Biel et al. [137]. Human skin samples were fixed by HPF, labeled with fluorescent dyes during freeze-substitution, and embedded in Epoxy resins. The entire resin-embedded specimen was studied by confocal laser scanning microscopy, a region of interest was chosen and marked at the surface of the resin block and consequently sectioned into ultrathin sections [138]. Alternatively, one could take a live cell sample exposed to NP, fix it by HPF and use it for further TEM imaging. Specific instrumentation, consumables and protocols were developed for this purpose [83]. For the whole lung, one might think of exposing the lung to NP (ideally by aerosol inhalation) to study the subsequent interactions by real-time lung microscopy [139] and fix the whole lung afterwards by vascular perfusion for further TEM investigations.

A solution of striking simplicity has surfaced recently. Based on the analysis of specific conjugated π-electrons, fluorochromes can be detected by improved EFTEM instrumentation. In this manner, Mhawi et al. [106] could reveal the fluorescent signal of doxorubicin in human breast cancer cells directly at the EM level without the need for further detection systems.

The potential of correlative light and electron microscopic approaches is enormous and their application in NP research may greatly enhance our understanding of the entering mechanisms, the intracellular trafficking and their downstream beneficial or toxic properties. The combination of the different advanced techniques described above offers a methodological armamentarium suited to answer many of the inherent questions currently discussed in NP research.

## 4. Focus C: Stereology

The significance of microscopic evaluations is directly associated with the extent and representativeness of the observations. Evaluation of the extent of a microscopic finding requires the use of unbiased quantitative methods which describe the structures in terms of number, length, area or volume. The representativeness of the observations is critical because the actual material that enters the microscopic stage is infinitesimally small in relation to what it is thought to represent. Therefore, the aim is to give every part of an organ or a cell culture an equal chance of being selected for the analysis. Stereology offers a wide range of unbiased methods that fulfil these requirements and are frequently used in pulmonary research, but their use in NP research is rare. There are, however, three basic scenarios that justify the use of stereology in NP research: (1) First, it is necessary to estimate the morphological changes that occur upon exposure to NP, e.g. the volume of fibrotic lesions in the lung or the number of inflammatory cells. (2) Second, changes in protein localization due to NP exposure can be estimated by relating immunogold particles to the relative volume of the organelles which results in relative labeling indices. (3) Third, the preferential distribution of NP within tissues or cells needs to be estimated and evaluated using appropriate statistical methods that are closely correlated to the methods for quantification of immunogold labeling. All of these methods require the use of sampling procedures that need to be followed consequently from the whole organ down to the microscopic test field under investigation. These methods include randomization of localization by systematic uniform random sampling [140] or fractionator sampling [141,142] and randomization of orientation using the isector [143] or the orientator [144]. The basic principles for applying unbiased stereology to lung research have been described in detail in some recent review articles [32,33].

Unfortunately, morphological changes associated with NP exposures are usually reported by micrographs showing "representative" lesions or are not investigated at all, a consequence from the fact that most studies focus on acute toxicity of NP rather than on chronic exposures. However, it will be necessary to assess these changes both in acute and chronic situations to evaluate the significance of the reported toxicological responses for pulmonary structural remodeling. To give some examples, stereology offers tools to investigate the number of cells or alveoli [145-147], the surface area of endocytosis-related cellular membranes (e.g. caveolae) or the gas exchange region [148], the volume of intracellular or intraalveolar surfactant components [149-151] and many more. For an overview of these methods, the reader may refer to the reviews of Ochs [32] and Weibel et al. [33].

The approaches to analyze immunogold or NP distributions using the comparison of observed and expected particle distributions in terms of indices (relative labelling index, RLI, [103], relative deposition index, RDI, [34]) and with chi-squared analysis have made it possible to distinguish between random and non-random particle distributions. Since RLI and RDI rely on the same basic principle they are only explained for the RDI and the reader may refer to Mayhew et al. [103] for further information on the RLI. The first step in the analysis of NP distribution within a certain tissue or cell is the definition of compartments within the structure of interest. For the lung, one may want to distinguish between airway lumen and wall, alveoli, epithelium, interstitium or endothelium of alveolar septa etc. For intracellular distributions different cellular compartments may be distinguished for the purpose of analysis. The second step is the gathering of a sufficient number of test fields by systematic uniform random sampling which serves to provide two sources of information: 1) The observed number of NP associated with each compartment can be estimated by counting the NP. It needs to be mentioned at this point that the probability a NP is contained in an ultrathin section increases with increasing particle size. If particles with a wide size distribution are used this may lead to a size-dependent bias due to oversampling of the larger sized particles. An unbiased approach of counting particles independent of their size is the use of disectors [145]. However, due to the small size of NP in relation to the thickness of the section it is usually not possible to employ physical disectors. The optical disector using ET may offer an alternative but for NP with a small size distribution and diameter it may be justified to count the particle profiles keeping in mind the chance of introducing a bias. 2) The relative volume of the compartments can be estimated by counting the number of points of a randomly superposed point grid hitting each compartment. This gives rise to the observed number of points. If the NP were distributed randomly within the tissue or cell then the observed number of NP associated with each compartment would be closely correlated with compartment size. This means that the observed number of points includes information about a hypothetical random NP distribution. This can be calculated from the numbers of observed particles and observed points and is called the expected number of particles. The relationship between the observed and expected number of particles can be expressed in terms of an index, the RDI, obtained by dividing the observed number by the expected number of particles for each compartment. If the RDI > 1 for a given compartment, it contains a greater number of NP than would be expected from the compartment size. If RDI < 1, a compartment contains less NP than would be expected from compartment size. And if RDI = 1 a compartment contains as many NP as would be expected from its size. The final step is to compare the observed and expected NP distributions in a statistically valid way using the chi-squared test. By using this test, we test the null-hypothesis of random distribution: "The observed and expected NP distributions are equal." For each compartment a partial chi-squared value is obtained. The larger the difference between the observed and expected number of particles the greater the partial chi-squared value will be and thus, the contribution of a compartment to the overall difference between observed and expected particle distribution as expressed by the sum of all partial chi-squared values, the total chi-squared value. The total chi-squared value indicates whether the null-hypothesis is rejected or accepted. If it is rejected, we can identify the preferential target of the NP when two criteria are met: RDI > 1 and the partial chi-squared value accounts for a substantial proportion of the total chi-squared value (say, 10% or more). For worked examples on synthetic and real sets of data the reader may refer to references [34,152] and to Table 2. Furthermore, a detailed discussion of the preconditions that have to be met is given in the original description [34].

**Table 2:** Synthetic data set in which nanoparticles are non-randomly distributed in different pulmonary tissue compartments. The hypothetical data were generated to provide an example of particles mainly taken up by macrophages or retained inside the lumen of the alveolar ducts/alveoli.

**Compartment**	**N**_O_	**P**	**N_E_**	**RDI**	**X^2^**	**Fraction of total X^2 ^[%]**
**Conductive airway (lumen)**	5	150	61.86	0.08	52.26	2.76
**Alveolus (lumen)**	527	1125	463.94	1.14	8.57	0.45
**Macrophages**	50	3	1.24	48.76	1781.45	94.22
**Epithelial cell**	12	38	15.67	0.77	0.86	0.05
**Interstitium**	13	34	14.02	0.93	0.07	0.004
**Endothelial cell**	12	38	15.67	0.77	0.86	0.05
**Residual**	0	113	46.6	0	46.6	2.46

**Total**	619	1501	619	1	1890.67	100

Note. With 6 degrees of freedom (7-1 compartments × 2-1 groups) and a total chi-squared value 1890.67 the null-hypothesis of random particle distribution has to be rejected with p < 0.001. The alveolar macrophages are the only compartment that fulfils the criteria for preferential particle localization: RDI > 1 and the partial chi-squared contributes substantially (more than 10%) to the non-randomness of the distribution, i.e. the total chi-squared [34, 103]. However, although the nanoparticles are preferentially located in the macrophages it is interesting to note that they deposit mainly in the alveolar region and are not found in the lumen of the blood vessels which are contained in the compartment residual. Abbreviations: N_O _= Number of observed particles; N_E _= Number of expected particles; P = Number of observed points; RDI = Relative deposition index; X^2 ^= Chi squared values.

## 5. Concluding remarks

This review article has shown that advanced TEM technology, ranging from specimen preparation to various analytical modes as well as to qualitative and quantitative interpretations of the ultrastructural NP localization is challenging and requires considerable resources and experience. Unawareness of the potentials and limitations of the applicability of a particular method contains the risk to lead to false conclusions. Therefore, the infrastructure required for TEM is often centralized in EM core facilities. If possible, one is well advised to ask for assistance at his/her local EM core facility at the earliest stage of planning. For example, with high-pressure freezing it is not possible to perform a proper systematic uniform random sampling as would be necessary for stereological purposes. On the other hand, fixation with high glutaraldehyde fixations might render a specimen unsuitable for immuno TEM purposes. Thus, the choice of the strategy for TEM analysis determines the mode of specimen fixation and sampling.

We have shown that advanced TEM holds great potential for NP research: High-resolution visualization of NP in a biological environment and quantitative evaluation of its localization will greatly enhance our understanding of the NP-cell interactions. Immunocytochemical TEM approaches combined with stereology will help to determine the effects of NP down to the protein level. Tomographic TEM provides the opportunity to increase our understanding of the three-dimensional interactions between NP and structures of the respiratory tract. Correlative light and electron microscopic approaches may combine live cell observations following NP exposure with subsequent detailed ultrastructral analyses.

We hope that this review contributes to a widespread application of advanced TEM techniques in NP research of the respiratory tract.

## Abbreviations

NP = Nanoparticles

TEM = Transmission electron microscopy

EFTEM = Energy filtered transmission electron microscopy

PFA = Paraformaldehyde

GA = Glutaraldehyde

HPF = High-pressure freezing

ET = Electron tomography

RLI = Relative labelling index

RDI = Relative deposition index

## Competing interests

The author(s) declare that they have no competing interests.

## Authors' contributions

CM planned the work presented in this article, performed parts of the experiments and electron microscopic analyses, and wrote major parts of the manuscript. BRR performed parts of the experiments, reviewed the manuscript critically and provided important intellectual content. DV performed parts of the experiments and electron microscopic analyses, wrote major parts of the manuscript and provided essential intellectual content. FB performed parts of the experiments and reviewed the manuscript critically. PG supervised the work on this article, made important suggestions, reviewed the manuscript critically and provided important intellectual content. MO participated in the development of this work, made important suggestions, reviewed the manuscript critically and provided essential intellectual content. All authors read and approved the manuscript.
